# Usefulness of Multi-Organ Point-of-Care Ultrasound as a Complement to the Decision-Making Process in Internal Medicine

**DOI:** 10.3390/jcm11082256

**Published:** 2022-04-18

**Authors:** Irene Casado-López, Yale Tung-Chen, Marta Torres-Arrese, Davide Luordo-Tedesco, Arantzazu Mata-Martínez, Jose Manuel Casas-Rojo, Esther Montero-Hernández, Gonzalo García De Casasola-Sánchez

**Affiliations:** 1 Department of Internal Medicine, Hospital Infanta Cristina, Parla, 28981 Madrid, Spain; irenemaria.casaso@salud.madrid.org (I.C.-L.); davide.tdsco@gmail.com (D.L.-T.); jm.casas@gmail.com (J.M.C.-R.); 2 Department of Internal Medicine, Hospital Universitario La Paz, 28046 Madrid, Spain; 3 Department of Medicine, Universidad Alfonso X, Villanueva de la Cañada, 28691 Madrid, Spain; 4 Department of Emergency Medicine, Hospital Universtario Fundación de Alcorcón, Alcorcón, 28922 Madrid, Spain; martatorresarrese@gmail.com (M.T.-A.); arantzazu.mata@salud.madrid.org (A.M.-M.); ggcasasolaster@gmail.com (G.G.D.C.-S.); 5 Department of Internal Medicine, Hospital Universitario Puerta de Hierro Majadahonda, IDIPHISA, 28222 Madrid, Spain; esthermhdez@hotmail.com

**Keywords:** point-of-care ultrasound, internal medicine, lung ultrasound, echocardiography, abdominal ultrasound

## Abstract

Accumulated data show the utility of diagnostic multi-organ point-of-care ultrasound (PoCUS) in the assessment of patients admitted to an internal medicine ward. We assessed whether multi-organ PoCUS (lung, cardiac, and abdomen) provides relevant diagnostic and/or therapeutic information in patients admitted for any reason to an internal medicine ward. We conducted a prospective, observational, and single-center study, at a secondary hospital. Multi-organ PoCUS was performed during the first 24 h of admission. The sonographer had access to the patients’ medical history, physical examination, and basic complementary tests performed in the Emergency Department (laboratory, X-ray, electrocardiogram). We considered a relevant ultrasound finding if it implied a significant diagnostic and/or therapeutic change. In the second semester of 2019, we enrolled 310 patients, 48.7% were male and the mean age was 70.5 years. Relevant ultrasound findings were detected in 86 patients (27.7%) and in 60 (19.3%) triggered a therapeutic change. These findings were associated with an older age (Mantel–Haenszel χ^2^ = 25.6; *p* < 0.001) and higher degree of dependency (Mantel–Haenszel χ^2^ = 5.7; *p* = 0.017). Multi-organ PoCUS provides relevant diagnostic information, complementing traditional physical examination, and facilitates therapy adjustment, regardless of the cause of admission. Multi-organ PoCUS to be useful need to be systematically integrated into the decision-making process in internal medicine.

## 1. Introduction

In the diagnostic and decision-making process in medicine, clinical history, and physical examination, based on inspection, palpation, percussion, and auscultation of different organs, are essential. Yet, in part because of the tremendous technological advances, clinicians’ interest and expertise in physical examination have diminished in recent decades.

Many studies have shown that point-of-care ultrasound (PoCUS) improves the diagnostic yield of physical examination. This is especially evident in cardiovascular examination (identification of heart valve lesions, estimate of central venous pressure), detection of pleural, pericardial, and abdominal free fluid, or the identification of splenomegaly or acute urinary retention [1,2,3,4]. It seems evident that PoCUS can revolutionize the way of examining patients since it complements and enhances traditional physical examination. Thus, together with inspection, palpation, percussion, and auscultation, it can become the fifth pillar of the physical examination [5,6].

The evaluation of some symptoms or signs, such as hypotension, dyspnea, or chest pain, multi-organ PoCUS is necessary in a structured protocol. As a result, many protocols have been published (i.e., *RUSH*, *SHoC*, *BLUE*, *FALLS*, *FEEL*, *SEARCH 8Es*, etc.) [7,8,9,10,11,12]. Most of these protocols rely on obtaining adequate views of the lung, heart, and abdomen (i.e., inferior vena cava or abdominal aorta assessment). In this approach, the concept of multi-organ PoCUS arises [13], demonstrating its usefulness in the emergency department (ED) regardless of the reason for consultation [14,15,16].

The aim of this study is to assess the usefulness of multi-organ PoCUS, including lung, heart, and abdomen, as a complement to medical history, physical examination, and initial complementary tests performed in the ED (blood tests, electrocardiograms, and X-rays). Providing an initial assessment in the first 24 h of hospital admission to an internal medicine ward.

## 2. Materials and Methods

We conducted a prospective, observational, and single-center study, at a secondary hospital, to assess the baseline characteristics, complementary test results, and multi-organ PoCUS results. The study is in accordance with the Declaration of Helsinki and was approved by the local Research Ethics Committee. Informed consent was obtained from each enrolled patient.

### 2.1. Patient Selection

Patients admitted to the internal medicine ward in the second semester of 2019 were screened. Patient eligibility was established by the availability of the sonographer to perform a multi-organ PoCUS in the first 24 h after admission, regardless of the reason for admission. Exclusion criteria included the use of any other imaging modality besides X-ray in the ED or previous admission in the last month (readmission). A sample of patients who met these inclusion criteria were enrolled and prospectively studied.

### 2.2. Epidemiological, Clinical, Laboratory and Radiological Data Assessment

Demographic data (age, sex, weight), medical history (comorbidities, medications), physical exam, laboratory tests (creatinine, urea, hemoglobin, white blood cells, platelets, D-dimer), electrocardiogram, variables correlated to therapy (type of medication, dose, duration), as well as variables correlated to treating physician gestalt, in the ED and internal medicine ward.

### 2.3. Ultrasound Data Collection

Internal medicine physicians with long-standing experience in PoCUS (more than 10 years of experience in performing and interpreting exams) performed the multi-organ PoCUS in all included patients.

**Figure 1:**
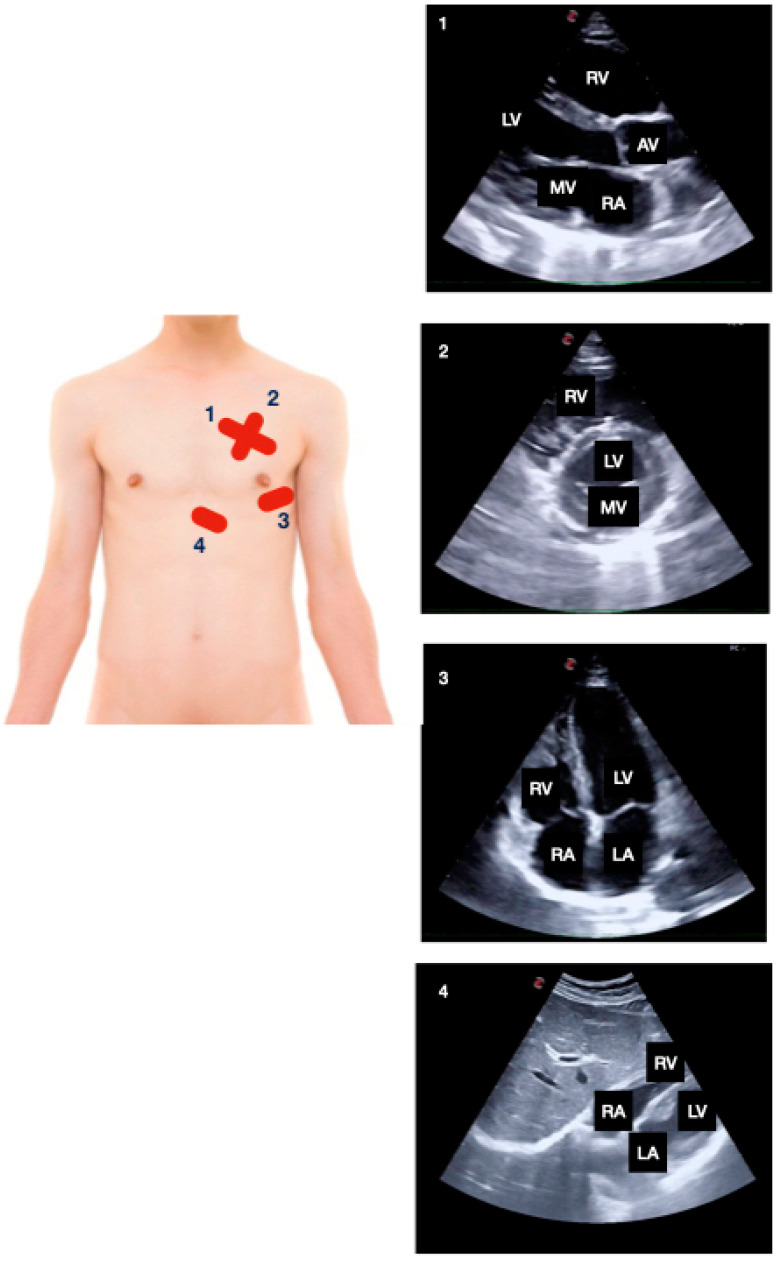
Focused cardiac ultrasound: (1) parasternal long and (2) short axis, (3) apical four chambers and (4) subxiphoid. AV: aortic valve, LA: left atrium, LV: left ventricle, MV: mitral valve, RA: right atrium, RV: right ventricle.

**Figure 2:**
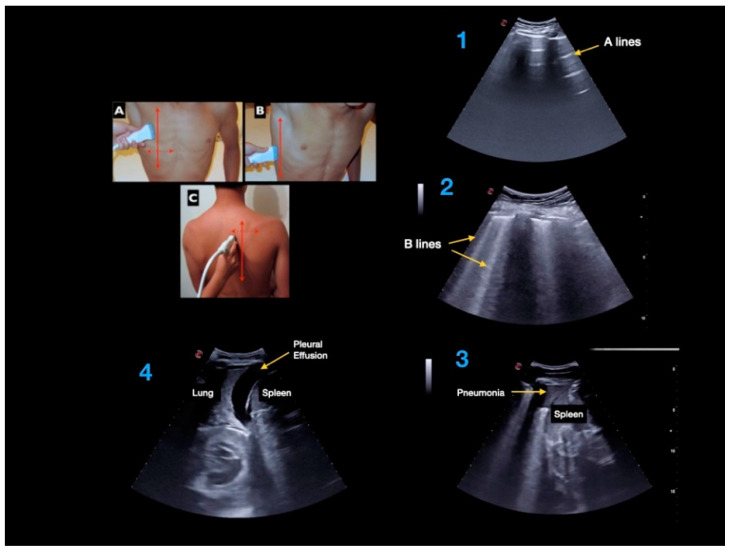
Lung ultrasound exam: (**A**) anterior, (**B**) lateral, and (**C**) posterior areas of both lungs. Lung ultrasound findings: (1) A-lines, (2) B-lines, (3) consolidation, and (4) pleural effusion.

**Figure 3:**
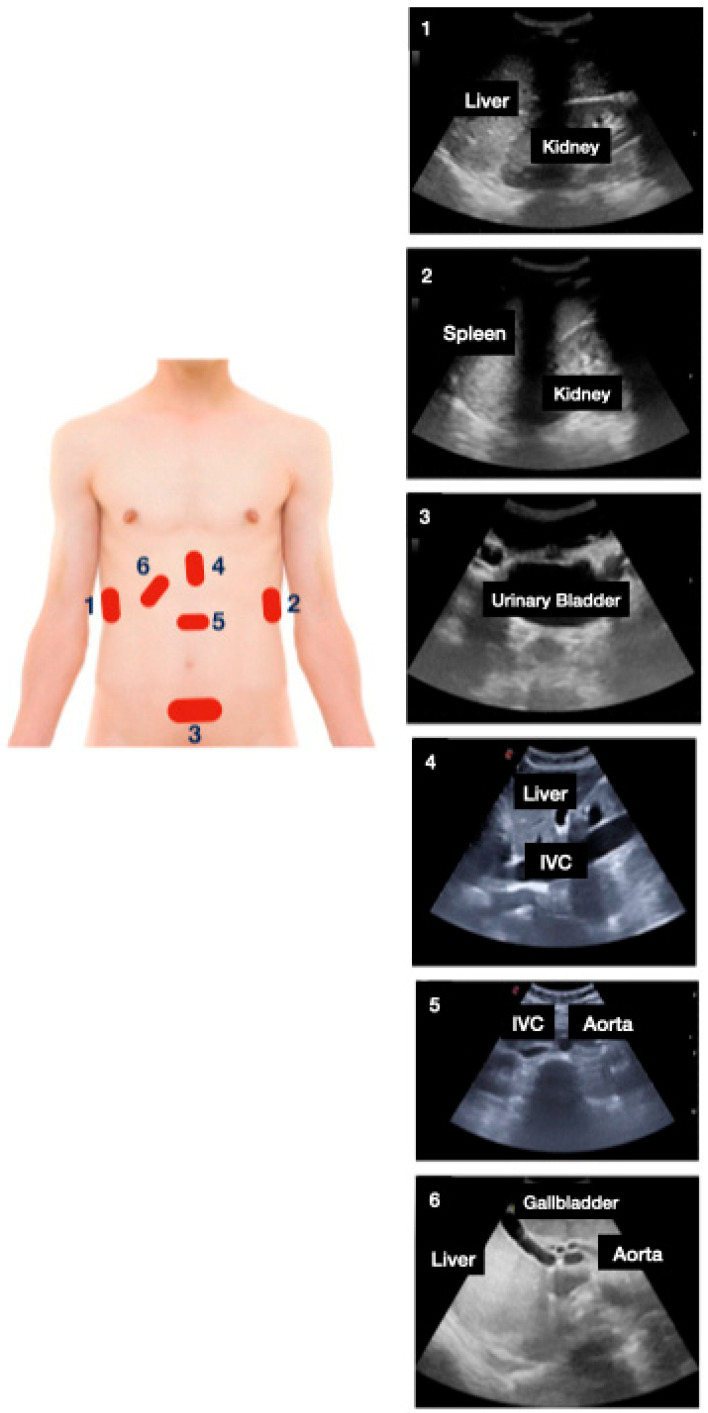
Abdominal *FAST*—*Focused Assessment with Sonography in Trauma*—protocol views: (1) perihepatic, (2) perisplenic, (3) pelvic, (4) subxiphoid longitudinal view, and (5) subxiphoid transverse view (IVC: inferior vena cava). Followed by hepatic and biliary protocol views: (6) right subcostal view.

The following multi-organ PoCUS protocol was performed using two-dimensional mode and color Doppler: focused cardiac ultrasound (Figure 1; subxiphoid, parasternal long and short axis, apical four chambers), lung ultrasound (Figure 2; anterior, lateral, and posterior areas of both lungs), abdominal *FAST*—*Focused Assessment with Sonography in Trauma*—protocol views (Figure 3; pericardial, perihepatic, perisplenic and pelvic), inferior vena cava (subxiphoid longitudinal view), abdominal aorta (subxiphoid transverse view), hepatic and biliary sonography (right subcostal view).

The study was performed using a MINDRAY M9 ultrasound system, with a linear probe (5–10 MHz), curvilinear probe (1–5 MHz), and phased array probe (1–5 MHz) (Mindray Medical España, Madrid, Spain).

After the multi-organ PoCUS exam, a report was issued with the most relevant findings. This information was shared with the treating physician and raised the question of whether these findings provided relevant information and/or implied substantial therapeutic changes.

### 2.4. Outcome Measures and Definitions

An electronic registry consisting of a database hosted on a web server was designed to help register the variables described above.

This electronic registry was Healthcare Insurance Portability and Accountability Act (HIPAA) compliant. Each investigator had an individual access code.

Each patient was follow-up during hospitalization (symptoms, final diagnosis, and date of discharge) and registered.

### 2.5. Statistical Analysis

We aimed to include patients in the same distribution as the background population in the internal medicine ward. A sample size of 300 patients was calculated according to the results obtained in a similar study carried out in the ED [17], which was thought to provide the capacity for subgroup analysis of changes in diagnosis and management and is believed to be feasible within the time frame. To our knowledge, no prior studies have provided sufficient knowledge for sample size estimation in an internal medicine ward.

Descriptive data are presented as actual numbers and percentages. Baseline characteristics are presented as the mean and standard deviation (SD) for continuous variables and count and proportions for categorical variables.

Our primary outcome was to determine the percentage of patients in whom multi-organ PoCUS modified the diagnosis and/or the therapy. To assess normality, a Shapiro–Wilk test was performed. We used a Pearson χ^2^ test and the Mantel–Haenszel χ^2^ for trend analysis. We assumed an α-value of 0.05 for two-sided hypothesis testing and a power of 80%. Analyses were conducted with the statistical Stata software v15.1 (StataCorp. 2017. Stata Statistical Software: Release 15. College Station, TX, USA: StataCorp LLC).

## 3. Results

**Figure 4:**
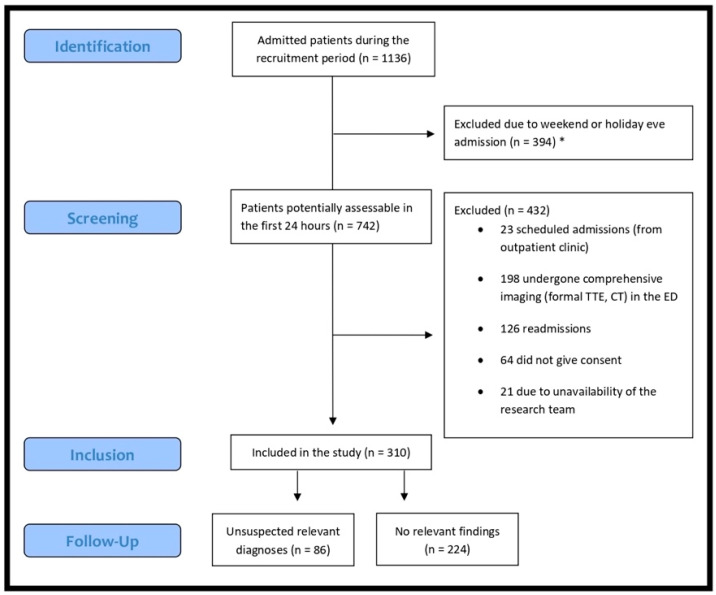
STROBE flow diagram. CT: computed tomography; ED: emergency department; TTE: transthoracic echocardiography. * Impossibility to perform the ultrasound in the first 24 h.

During the second semester of 2019, a total of 1136 patients were admitted to the internal medicine ward and screened for eligibility. Of this total, 310 were finally enrolled in the study. Reasons for exclusion are detailed in Figure 4.

Baseline demographics, patient characteristics, and physical exam of the patients included in the study are summarized in Table 1. The mean age was 70.5 years (SD 18), and nearly half were male (149, 48.7%). Most of them were overweight (mean body mass index 27.6 kg/m^2^, SD 5.6) and were at least minimally dependent (mean Barthel index 78, SD 29). The patients were normotensive with a normal oxygen saturation (94%, SD 3).

Table 2 shows the main reasons for admission before performing the multi-organ PoCUS. The most common causes for admission were lower respiratory tract infection (29.3%), acute heart failure (16.8%), and urinary tract infection (11.3%).

After performing the multi-organ PoCUS, unsuspected relevant diagnoses were detected in 86 patients (27.7%), and this conditioned a therapeutic modification in 60 patients (19.3%). Table 3 shows the unsuspected diagnoses detected by multi-organ PoCUS. The total number of unsuspected diagnoses was greater than 86 since more than one diagnosis was detected in 24 patients (two diagnoses in 21 patients, three in one patient, and four diagnoses in another).

The finding of a relevant diagnosis was associated with age; the older the age (in quintiles) the greater the probability of detecting an unsuspected relevant diagnosis (Mantel–Haenszel χ^2^ = 25.6; *p* < 0.001) as shown in Table 4. Of the 89 patients in whom a relevant diagnosis was detected, 69 (77.5%) were older than 65 years (χ^2^ Pearson = 5.39; *p* = 0.021). We also found an association between the degree of dependency and the detection of a relevant diagnosis (Mantel–Haenszel χ^2^ = 5.7; *p* = 0.017).

## 4. Discussion

Although chest CT might offer a more accurate way to diagnose COVID-19 lung involvement, due to the scale of the pandemic, its routine use for this purpose is not available in most hospitals. Therefore, alternatives such as chest X-ray and lung ultrasound should be explored. Several studies have shown that lung ultrasound has greater sensitivity than chest X-ray [13] and has a good correlation with chest CT [5,14].

In this prospective observational study, we have demonstrated the enormous utility of multi-organ PoCUS in the diagnostic process (decision-making process) of patients admitted to an internal medicine ward. In approximately one in four patients, relevant alternative diagnoses were established and in one in five it caused a therapeutic modification. This high percentage of patients in whom multi-organ PoCUS was beneficial may seem too high, but similar results have been observed in ED [15,16,17] and intensive care unit [18,19,20] patients. In a recent systematic review [21] about the usefulness of PoCUS in patients admitted to an internal medicine ward, PoCUS findings allowed a therapeutic adjustment in 20–40% of the patients and provided an unsuspected diagnosis in 34% of patients, similar to our study results. Moreover, some studies suggest that PoCUS can help reduce hospital length of stay [22,23]. Yet, it has not been possible to prove that PoCUS could reduce mortality.

This group believes that one of the explanations to achieve these results is to systematize the way PoCUS is performed, similar to traditional physical examination. It is essential to perform a multi-organ PoCUS within a protocol, like the one we followed, with systematic views of the lung, heart, and abdomen [14]. After adequate training, our proposed multi-organ PoCUS exam can be performed in less than 15 min.

Nevertheless, analyzing our results, we can make the following considerations regarding each pathology:

1. Dyspnea is a very common reason for admission and multiorgan PoCUS might be especially useful [24,25,26,27]. In fact, acute heart failure and the detection of a significant cardiac abnormality (valvular heart disease, left ventricular systolic dysfunction, pulmonary hypertension) have accounted for a very high percentage of unsuspected diagnoses made by PoCUS.
2. The high prevalence of relevant cardiac abnormalities, especially significant valve disease, is related to aging and frequently seen in admitted patients to the internal medicine wards.
3. Lung ultrasound has allowed the diagnosis of a significant percentage of pneumonia and complicated pleural effusion. Especially in older patients, chest X-ray might not be accurate, and it can be difficult to visualize pneumonia located in the lower posterior regions of the lungs or whether a pleural effusion is complicated (i.e., presence of fibrous tracts) [28,29].
4. Acute urinary retention is relatively common, and predominantly affects older men.
5. It is important to explore the abdominal aorta in the presence of cardiovascular risk factors (i.e., smoking) [30].
6. Excessive volume intake can lead to a systemic venous congestion in a short time, especially in malnourished individuals (i.e., low albumin levels). Lung ultrasound can aid in detecting signs of early congestion.
7. As expected, older patients have higher probabilities of exhibiting unsuspected diagnoses through multi-organ PoCUS. The same happens with the level of dependency, although it is very likely that age might act as a cofounding factor.

In our study, we detected very few patients with unsuspected thromboembolic disease. This is because most of these patients’ diagnoses are made in the ED by CT scan and so are excluded from our study.

This study contributes to the general knowledge of the prevalence of ultrasound findings in hospitalized patients, which will be important for any clinician performing an ultrasound. Using basic multi-organ PoCUS examinations as a screening tool may lead to the early detection of occult pathology and early treatment. Translating this knowledge is paramount to understanding the pre- and post-test probability of presenting relevant findings of any admitted patient.

To the best of our knowledge, our study is the first to assess the impact of a protocolized and systematic multi-organ PoCUS in an internal medicine ward. A strength of our real-life practice study is that, as expected, the cohort is heterogeneous, which allows us to emphasize the fact that multi-organ PoCUS is exceedingly useful, as suggested by previous studies, in other clinical settings.

### Limitations

We acknowledge some study limitations. First, the study was carried out in a single hospital center and in which a small number of internist sonographers participated. It is a known limitation that PoCUS is dependent on competent operators performing the examination, and the main barrier to its expansion. Thus, this may reduce the external validity of the study. Another limitation is that emergency physicians’ confidence also influences when they establish the initial diagnostic suspicion.

Thus, for this purpose, we suggest our study can be considered hypothesis generating and the conclusions must be contrasted with larger studies.

## 5. Conclusions

In conclusion, multi-organ PoCUS facilitates unsuspected diagnoses in a high proportion of patients admitted to an internal medicine ward, regardless of the initial cause of admission. This determines changes in treatment in many of these patients. Multi-organ PoCUS should be systematically integrated into the decision-making process in internal medicine.

## Figures and Tables

**Table 1:** Demographics, clinical characteristics, and ultrasound severity classification of patients included.

Demographics	
Gender (male)—N (%)	149 (48.7)
Age (years) mean (SD)	70.5 (18)
Past Medical History	N (%)
Diabetes mellitus—N (%)	32 (10.3)
Body mass index (kg/m^2^) mean (SD)	27.6 (5.6)
Smoking habit—N (%)	59 (19.2)
Excessive alcohol consumption (>20 g/day)—N (%)	32 (10.3%)
Barthel index mean (SD)	78 (29)
Moderate to high disability (Barthel index < 60)—N (%)	86 (27.7%)
Physical Exam	
SBP (mmHg) mean (SD)	130 (21)
DBP (mmHg) mean (SD)	71 (14)
Heart rate (bpm) mean (SD)	82 (16)
SO_2_ (%) mean (SD)	94 (3)

DBP: diastolic blood pressure; SBP: systolic blood pressure; SD: standard deviation.

**Table 2:** Main reason for admission before multi-organ point-of-care ultrasound (N = 310) *.

Reason for Admission	N (%) *
Lower respiratory tract infection	91 (29.3)
Acute heart failure	52 (16.8)
UTI	35 (11.3)
COPD exacerbation	28 (9)
Infectious diseases (non-respiratory or UTI)	11 (3.5)
Chronic respiratoria exacerbation (non-COPD)	9 (2.9)
VTE disease	8 (2.6)
Gastrointestinal pathology (hepatitis, cholecystitis, cholangitis)	7 (2.3)
Cardiac arrythmia	4 (1.3)
Cerebrovascular disease	3 (1)
Other diagnosis	92 (29.6)

COPD: chronic obstructive pulmonary disease; UTI: urinary tract infection; VTE: venous thromboembolism. * The total sum of diagnostic reasons (340) exceeds the total number of patients included (310) since some of the patients had more than one reason for admission.

**Table 3:** Relevant unsuspected diagnoses detected after multi-organ point-of-care ultrasound (N = 310).

Final Diagnosis	N (%)
Significant valvular disease (unknown)	15 (4.8)
Heart failure	14 (4.5)
Pneumonia	14 (4.5)
Acute urinary retention	10 (3.2)
Congestive status	9 (2.9)
Severe pulmonary hypertension (unknown)	8 (2.6)
Moderate to severe systolic dysfunction (unknown)	5 (1.6)
Abdominal aorta aneurism	5 (1.6)
Hydronephrosis	7 (2.2)
Lung interstitial disease (unknown)	4 (1.3)
Complicated pleural effusion (empyema)	4 (1.3)
Moderate to severe pericardial effusion	4 (1.3)
Metastatic liver	3 (0.9)
Oher diagnosis	10 (3.2)

**Table 4:** Risk of relevant unsuspected diagnosis by multi-organ point-of-care ultrasound stratified by age (N = 310).

Age Stratification	N (%)	Unsuspected Diagnosis	Risk (%)	Relative Risk	95% Confidence Interval	
<56	64 (20.6)	11	17.1	1		
56–69	65 (21.0)	15	23.07	1.32	0.7	2.65
70–79	62 (20.0)	17	27.42	1.57	0.8	3.08
79–87	59 (19.0)	16	27.11	1.55	0.8	3.07
87–100	60 (19.4)	30	50	2.91	1.61	5.27

## Data Availability

The authors confirm that the data supporting the findings of this study are available from the corresponding author, upon reasonable request.
